# Supplementary material for: Pyruvate Dehydrogenase Kinase 1 inhibition mediated oxidative phosphorylation enhancement in cartilage promotes osteoarthritis progression
Source: BMC Musculoskelet Disord. 2023 Jul 20;24:597. doi: 10.1186/s12891-023-06585-6 (PMC10357736; doi:10.1186/s12891-023-06585-6)
Supplement: Supplementary file 1 — Additional file 1. [file 12891_2023_6585_MOESM1_ESM.zip › MF.Gsea.1653627316242/GOMF_TRANSFORMING_GROWTH_FACTOR_BETA_RECEPTOR_BINDING.html]

Details for gene set GOMF\_TRANSFORMING\_GROWTH\_FACTOR\_BETA\_RECEPTOR\_BINDING[GSEA]

|  || Dataset | 55457\_collapsed\_to\_symbols.cls.cls #OA\_versus\_normal.cls.cls #OA\_versus\_normal\_repos |
| Phenotype | cls.cls#OA\_versus\_normal\_repos |
| Upregulated in class | OA |
| GeneSet | GOMF\_TRANSFORMING\_GROWTH\_FACTOR\_BETA\_RECEPTOR\_BINDING |
| Enrichment Score (ES) | 0.39969704 |
| Normalized Enrichment Score (NES) | 1.3079917 |
| Nominal p-value | 0.13574661 |
| FDR q-value | 0.39937645 |
| FWER p-Value | 1.0 |
Table: GSEA Results Summary

  

Fig 1: Enrichment plot: GOMF\_TRANSFORMING\_GROWTH\_FACTOR\_BETA\_RECEPTOR\_BINDING      
 Profile of the Running ES Score & Positions of GeneSet Members on the Rank Ordered List

  

| SYMBOL | TITLE | RANK IN GENE LIST | RANK METRIC SCORE | RUNNING ES | CORE ENRICHMENT || 1 | SMAD2 | SMAD family member 2 [Source:HGNC Symbol;Acc:HGNC:6768] | 275 | 0.768 | 0.1504 | Yes |
| 2 | SNX6 | sorting nexin 6 [Source:HGNC Symbol;Acc:HGNC:14970] | 769 | 0.576 | 0.2408 | Yes |
| 3 | RASL11B | RAS like family 11 member B [Source:HGNC Symbol;Acc:HGNC:23804] | 1983 | 0.363 | 0.2277 | Yes |
| 4 | FKBP1A | FKBP prolyl isomerase 1A [Source:HGNC Symbol;Acc:HGNC:3711] | 2293 | 0.328 | 0.2769 | Yes |
| 5 | LEFTY2 | left-right determination factor 2 [Source:HGNC Symbol;Acc:HGNC:3122] | 2406 | 0.314 | 0.3383 | Yes |
| 6 | TGFB1 | transforming growth factor beta 1 [Source:HGNC Symbol;Acc:HGNC:11766] | 2490 | 0.303 | 0.3997 | Yes |
| 7 | SMAD6 | SMAD family member 6 [Source:HGNC Symbol;Acc:HGNC:6772] | 3460 | 0.206 | 0.3705 | No |
| 8 | SMAD3 | SMAD family member 3 [Source:HGNC Symbol;Acc:HGNC:6769] | 4061 | 0.150 | 0.3574 | No |
| 9 | INHBC | inhibin subunit beta C [Source:HGNC Symbol;Acc:HGNC:6068] | 4788 | 0.088 | 0.3207 | No |
| 10 | AMH | anti-Mullerian hormone [Source:HGNC Symbol;Acc:HGNC:464] | 5042 | 0.070 | 0.3166 | No |
| 11 | TGFBR1 | transforming growth factor beta receptor 1 [Source:HGNC Symbol;Acc:HGNC:11772] | 5280 | 0.050 | 0.3093 | No |
| 12 | LEFTY1 | left-right determination factor 1 [Source:HGNC Symbol;Acc:HGNC:6552] | 5935 | -0.000 | 0.2585 | No |
| 13 | TGFB3 | transforming growth factor beta 3 [Source:HGNC Symbol;Acc:HGNC:11769] | 6293 | -0.027 | 0.2368 | No |
| 14 | TGFBR3 | transforming growth factor beta receptor 3 [Source:HGNC Symbol;Acc:HGNC:11774] | 6868 | -0.069 | 0.2076 | No |
| 15 | TGFB2 | transforming growth factor beta 2 [Source:HGNC Symbol;Acc:HGNC:11768] | 6985 | -0.079 | 0.2162 | No |
| 16 | ENG | endoglin [Source:HGNC Symbol;Acc:HGNC:3349] | 7213 | -0.097 | 0.2202 | No |
| 17 | SMAD7 | SMAD family member 7 [Source:HGNC Symbol;Acc:HGNC:6773] | 7231 | -0.098 | 0.2407 | No |
| 18 | TGFBRAP1 | transforming growth factor beta receptor associated protein 1 [Source:HGNC Symbol;Acc:HGNC:16836] | 7347 | -0.106 | 0.2554 | No |
| 19 | BAMBI | BMP and activin membrane bound inhibitor [Source:HGNC Symbol;Acc:HGNC:30251] | 7903 | -0.148 | 0.2453 | No |
| 20 | TGFBR2 | transforming growth factor beta receptor 2 [Source:HGNC Symbol;Acc:HGNC:11773] | 8241 | -0.174 | 0.2579 | No |
| 21 | USP15 | ubiquitin specific peptidase 15 [Source:HGNC Symbol;Acc:HGNC:12613] | 8653 | -0.205 | 0.2718 | No |
| 22 | FERMT2 | FERM domain containing kindlin 2 [Source:HGNC Symbol;Acc:HGNC:15767] | 9349 | -0.259 | 0.2757 | No |
Table: GSEA details [plain text format]

  

Fig 2: GOMF\_TRANSFORMING\_GROWTH\_FACTOR\_BETA\_RECEPTOR\_BINDING      
 Blue-Pink O' Gram in the Space of the Analyzed GeneSet

  

Fig 3: GOMF\_TRANSFORMING\_GROWTH\_FACTOR\_BETA\_RECEPTOR\_BINDING: Random ES distribution      
 Gene set null distribution of ES for **GOMF\_TRANSFORMING\_GROWTH\_FACTOR\_BETA\_RECEPTOR\_BINDING**
